# Structures of SRP54 and SRP19, the Two Proteins that Organize the Ribonucleic Core of the Signal Recognition Particle from *Pyrococcus furiosus*

**DOI:** 10.1371/journal.pone.0003528

**Published:** 2008-10-27

**Authors:** Pascal F. Egea, Johanna Napetschnig, Peter Walter, Robert M. Stroud

**Affiliations:** 1 Department of Biochemistry and Biophysics, University of California San Francisco, San Francisco, California, United States of America; 2 Laboratory of Cell Biology and Howard Hughes Medical Institute, The Rockefeller University, New York, New York, United States of America; Massachusetts Institute of Technology, United States of America

## Abstract

In all organisms the Signal Recognition Particle (SRP), binds to signal sequences of proteins destined for secretion or membrane insertion as they emerge from translating ribosomes. In Archaea and Eucarya, the conserved ribonucleoproteic core is composed of two proteins, the accessory protein SRP19, the essential GTPase SRP54, and an evolutionarily conserved and essential SRP RNA. Through the GTP-dependent interaction between the SRP and its cognate receptor SR, ribosomes harboring nascent polypeptidic chains destined for secretion are dynamically transferred to the protein translocation apparatus at the membrane. We present here high-resolution X-ray structures of SRP54 and SRP19, the two RNA binding components forming the core of the signal recognition particle from the hyper-thermophilic archaeon *Pyrococcus furiosus* (*Pfu*). The 2.5 Å resolution structure of free *Pfu*-SRP54 is the first showing the complete domain organization of a GDP bound full-length SRP54 subunit. In its *ras*-like GTPase domain, GDP is found tightly associated with the protein. The flexible linker that separates the GTPase core from the hydrophobic signal sequence binding M domain, adopts a purely α-helical structure and acts as an articulated arm allowing the M domain to explore multiple regions as it scans for signal peptides as they emerge from the ribosomal tunnel. This linker is structurally coupled to the GTPase catalytic site and likely to propagate conformational changes occurring in the M domain through the SRP RNA upon signal sequence binding. Two different 1.8 Å resolution crystal structures of free *Pfu*-SRP19 reveal a compact, rigid and well-folded protein even in absence of its obligate SRP RNA partner. Comparison with other SRP19•SRP RNA structures suggests the rearrangement of a disordered loop upon binding with the RNA through a reciprocal induced-fit mechanism and supports the idea that SRP19 acts as a molecular scaffold and a chaperone, assisting the SRP RNA in adopting the conformation required for its optimal interaction with the essential subunit SRP54, and proper assembly of a functional SRP.

## Introduction

In all living cells the signal recognition particle (SRP) recognizes nascent polypeptides destined for secretion or membrane insertion as they emerge from translating ribosomes [1], [2]. As SRP binds to signal sequences emerging from the ribosomes, the resulting complex composed of the SRP and the ribosome-nascent chain complex is then targeted towards the membrane through the GTP-dependent interaction with the membrane-associated SRP receptor (SR also named FtsY in bacteria). Both SRP and SR contain GTPase domains. Their tight association triggers the reciprocal activation of GTP hydrolyses that govern docking and release of the ribosome-nascent chain to the translocon and recycling of the SRP. Although the SRP pathway is evolutionarily conserved, the composition of the SRP and its receptor SR varies widely. All SRPs from bacteria to eukaryotes and archaea, with the exception of chloroplastic SRPs, require the essential SRP RNA to function. SRP RNA has been shown to play a central role in the protein targeting reaction by catalyzing the interaction between SRP and its receptor [3] but also, albeit to a lesser extent, in accelerating GTP hydrolysis in the SRP•SR complex once formed [4].

**Figure 1:**
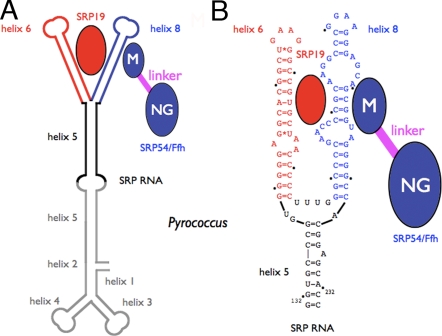
The archeal Signal Recognition Particle. (A) Simplified schematic of the archaeal signal recognition particle from *Pyrococcus furiosus*. (B) The sequence and organization of the core of the SRP RNA are shown with helices 6 and 8, the respective binding sites for the proteins SRP19 and SRP54/Ffh. For SRP54/Ffh the M domain, responsible for both SRP RNA and signal sequence recognition, is connected to the NG domain with the GTPase activity, through a flexible linker (in magenta). Although the NG of SRP54 domain has also been shown to interact loosely with the core of the SRP RNA, for the sake of clarity this is not represented on this schematic.

In eukaryotes, the SRP contains six proteins (SRP68/SRP72, SRP9/SRP14, SRP54 and SRP19) and a 300-nucleotide RNA. Most bacterial systems display a simplest organization with a shorter RNA (about 113 nucleotides) and a single protein subunit Ffh, the homologue of SRP54. Archeal SRPs contain an SRP RNA of similar size and fold to that in eukaryotes but only two proteins, SRP54 and SRP19 (**Figure 1A**). Thus, archaeal SRPs represent a more streamlined version of the eukaryotic homologues and provide an opportunity to explore an increased repertoire in structural and biophysical terms.

Although the overall composition of the SRP systems differ, the central ribonucleoprotein core and the general mechanism of GTP-dependent targeting are highly conserved. Since SRP54 is the only protein subunit conserved in all SRPs, it represents the key component in protein targeting. It is essential for signal sequence recognition and binding at the ribosome, and for the GTP-dependent interaction with SR its cognate receptor. This interaction determines proper transfer of the ribosome-nascent chain complex to the protein-translocating channel in the membrane. The dual function is supported by its multi-domain structure composed of an N-terminal domain (a four α-helix bundle), a central GTPase domain (with a *ras*-like fold) and the C-terminal M domain (for methionine rich). While the N and G domains associate together to constitute a structural and functional catalytic core, the M domain is responsible for the promiscuous recognition of the diverse signal sequences and binding to the helix 8 of the SRP RNA. A flexible linker relates the catalytic NG core to the signal peptide and RNA-binding M domain (**Figure 1B**).

SRP19 is found only in archaeal and eukaryotic SRPs and is involved in the proper assembly of the functional ribonucleoprotein complex. It binds primarily to helix 6 of the SRP RNA and contributes to the proper folding of the SRP RNA by bridging together and stabilizing [5], [6] helices 6 and 8 (**Figure 1B**). *In vitro* reconstitution with purified archeal components from *Archaeoglobus fulgidus* [7], [8], *Methanococcus jannaschii* [9], *Haloferax volcanii* [10], or *Pyrococcus furiosus* [11] have shown binding of SRP54 to SRP RNA even in absence of SRP19. SRP19 appears to be a dispensable component in the SRP from the archaeon *Haloferax* [12]. Although SRP54 shows inherent affinity for SRP RNA, these studies showed that SRP19 is required for high-affinity binding. In eukaryotes, the situation is slightly more complex. Due to cellular compartmentalization, SRP functions in the cytoplasm. Studies with yeast [13], [14] and mammalian [15]–[17] cells support a model in which all SRP proteins, except SRP54, are imported into the nucleolus/nucleus for assembly with the SRP RNA. The resulting “pro-particle” is exported back to the cytoplasm where it incorporates SRP54 into a fully functional ribonucleic particle. Despite these differences, the intrinsic structural features of the signal recognition particle are so robustly conserved throughout evolution that ribonucleic particles reconstituted *in vitro* by mixing bacterial, archaeal, and, eukaryotic components are functional.

## Results and Discussion

We cloned, expressed and determined the corresponding crystal structures of SRP54 and SRP19, the two RNA binding proteins that constitute the signal recognition particle of the hyperthermophilic archaeon *Pyrococcus furiosus*. Both SRP54 and SRP19 structures were solved *de novo* using anomalous dispersion phasing methods (**Table 1** **and** **Material and Methods**), and are therefore not biased towards any of their previously solved homologues. In both cases the proteins were crystallized in absence of RNA for future comparison with RNA bound structures. The two free subunits *Pfu*-SRP54 and *Pfu*-SRP19 proved to be remarkably robust; their purification involved a heating step at 75°C for 45 minutes and the corresponding purified proteins yielded well-ordered crystals that diffracted to high resolution. Prior to this work, *Pfu*-SRP54 and its associated *Pfu*-SRP RNA have been partially characterized *in vitro* [11]. Although the full length *Pfu*-SRP RNA is 314 nucleotides long, the recombinant protein expressed in *E.coli* was shown to bind tightly (Kd = 18 nM) to a conserved region corresponding to the so-called core RNA (**Figure 1B**).

**Table 1:** X-ray data collection and structure refinement statistics.

Structure	*Pfu* –SRP54 with GDP	*Pfu*-SRP19	*Pfu*-SRP19
PDB ID	3DM5	3DLU	3DLV
data set	ALS011204-030605	ALS031005	ALS080506
**data statistics**			
wavelength	1.11589 Å/0.97949 Å	0.92004 Å	0.97949 Å
phasing method	MR/Se-SAD	Br-SAD	Se-SAD/MR
space group and cell dimensions	P4_2_2_1_2	P2_1_	C222_1_
	a = 127.0 Å	a = 44.1 Å	a = 35.3 Å
	c = 186.9 Å	b = 79.7 Å	b = 116.1 Å
		c = 60.5 Å	c = 84.5 Å
		β = 107.7°	
ASU content	2 molecules	4 molecules	2 molecules
Solvent content	69%	42%	34%
resolution limits (last shell)	50-2.5 Å (2.6-2.5 Å)	50-1.8 Å (1.85-1.79 Å)	50-1.87 Å (1.94-1.87 Å)
unique reflections	53,350 (3,675)	37,519 (3,075)	14,745 (1,411)
redundancy	4.1 (3.4)	4.1 (3.6)	7.9 (6.2)
completeness	99.5% (98.4%)	99.8% (98.3%)	99.8% (98.3%)
*I/σ(I)*	12.7 (1.7)	14.9 (2.7)	22.3 (2.0)
*R_sym_*	5.6% (76.3%)	6.9% (42.7%)	5.2% (51.3%)
**refinement statistics**			
resolution range	64.7-2.5 Å	42.0-1.79 Å	47.8-1.87 Å
reflections used work (test)	50,650 (2,000)	34,397 (1,987)	12,588 (1,399)
*R_free_/R_fac_*	25.9%/22.2%	22.5%/19.1%	25.8%/19.9%
overall figure of merit	0.890	0.913	0.916
overall B_wilson_	55 Å^2^	21 Å^2^	21 Å^2^
B factor protein atoms	6,388 atoms, 66 Å^2^	2,997 atoms, 28 Å^2^	1,531 atoms, 29 Å^2^
B factor ligand atoms	2 GDPs, 45 and 43 Å^2^	no ligands	no ligands
	12 sulfates, 99 Å^2^	39 bromides, 38 Å^2^	
	2 acetates, 75 Å^2^	1 malonate, 38 Å^2^	
B factor solvent atoms	142 waters, 49 Å^2^	228 waters, 38 Å^2^	89 waters, 33 Å^2^
r.m.s.d. bonds	0.008 Å	0.007 Å	0.004 Å
r.m.s.d. angles	1.168°	1.030°	0.812°
Ramachandran Analysis			
residues in preferred regions	92.9%	99.7%	98.4%
residues in allowed regions	5.5%	0.3%	1.6%
outliers	1.6%	0%	0%

MR indicates phasing using molecular replacement. Br-SAD and Se-SAD respectively indicate phasing performed using single wavelength anomalous dispersion of bromine or selenium. ASU stands for asymmetric unit.

r.m.s.d is the root-mean square deviation from ideal geometry.

*R_sym_* = Σ_hkl_Σ_i_ |*I*_hkl,i_−〈*I*_hkl,i_〉|/Σ_hkl_Σ_i_ |*I*_hkl,i_| where 〈*I*_hkl,i_〉 is the average intensity of the multiple hkl, i observations for symmetry-related reflections.

*R_cryst_* = Σ|*F*_obs_−*F*_calc_|/Σ|*F*_obs_|. *F*_obs_ and *F*_calc_ are observed and calculated structure factors, *R_free_* is calculated from a set of randomly chosen 5 to 10% of reflections, and *R_cryst_* is calculated over the remaining 90 to 95% of reflections.

### SRP54 from *Pyrococcus furiosus* adopts an extended conformation

**Figure 2:**
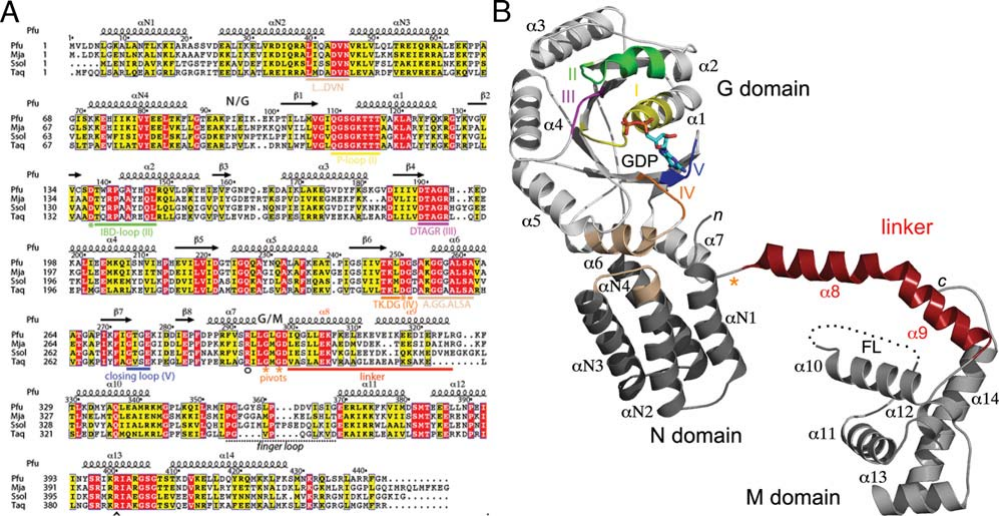
The SRP54 from *Pyrococcus furiosus*. (A) Sequence alignment of Ffh/SRP54 full-length proteins of known structure including *Pyrococcus furiosus*, *Thermus aquaticus*, *Methanococcus jannaschii* and *Sulfolobus solfataricus*. α-helices and conserved motifs of the SRP/SR-GTPase subfamily are labeled. The N, G and M domains are indicated, as is the linker region between the G and M domains. (B) Overall structure of the monomer of *Pfu*-SRP54. The secondary structure elements are indicated. The bound-GDP nucleotide is represented in sticks and the disordered finger loop (FL) schematized as a dashed line. The distance between the end of the NG domain (Leu296 at the C-terminus of helix α7) and the N-terminus of the M domain (Gly326 at the C-terminus of helix α9) is 44 Å.

This is the third X-ray structure of a free full-length SRP54/Ffh[18], [19], not including the only structure of full length SRP54 in complex with the core SRP RNA and SRP19 [20]. However, in the first structure of full-length Ffh from the bacteria *Thermus aquaticus* reported from our laboratory, [18], the GM-linker was only partially ordered and because there were three molecules in the asymmetric unit, there was an ambiguity regarding the relative orientation between the NG catalytic core and the M domain. The second structure of SRP54, from the archaeon *Sulfolobus solfataricus* [19], despite its moderate resolution (about 4 Å), revealed this linker and a subset of hydrophobic interactions between somewhat conserved residues of the N and M domains. The crystal structure of *Pfu*-SRP54 bound to GDP reported here reveals a new conformation for the essential SRP-GTPase where the M domain stands as a physically separated domain connected to the NG domains through the G to M linker that adopts a purely α-helical conformation comprising helices α8 and α9 (**Figures 2** **and** **3A**). A similar linker conformation was also observed in the *Ssol*-SRP54 structure (**Figure 4A**). In this relative arrangement the distance between the C-terminus of the G domain (residue Gly296 at the end of helix α7) and the N-terminus of the M domain (residue Gly 326 at the end of helix α9) is about 44 Å. The NG domain is well defined with GDP bound at the active site.

**Figure 3:**
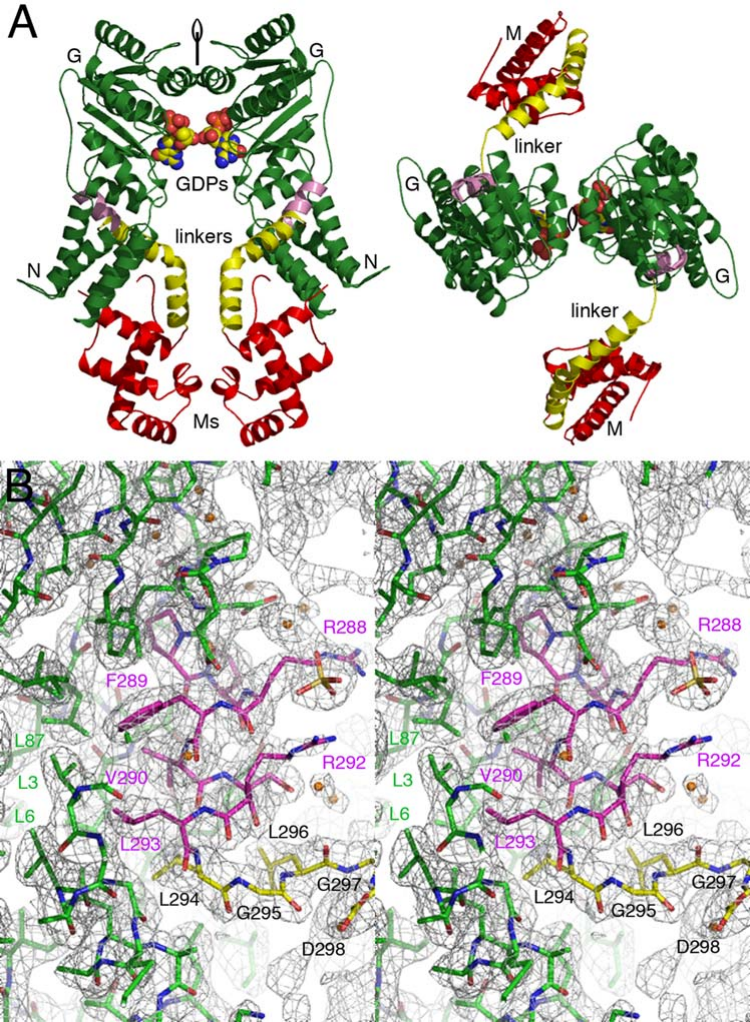
An extended conformation of the linker in *Pfu*-SRP54. (A) Arrangement of the non-crystallographic dimer of *Pfu*-SRP54•GDP in the tetragonal asymmetric unit. The NG catalytic cores, linkers and M domains are labeled and respectively colored in green, yellow and red. The non-crystallographic two-fold axis is represented on both views. The two views are perpendicularly related. (B) Stereo view showing the final 2.5 Å resolution *2mFo-DFc* Fourier difference likelihood-weighted electron density map contoured at 1.2σ in the linker region between the NG and M domains. The strictly conserved residues involved in GDP binding are labeled and the hydrogen bonds drawn. The conserved residues in the R_292_XLGXGD_298_ motif of the GM linker are labeled. Residues from the NG domain, its C-terminal α7 helix and the linker are respectively colored in green, pink and yellow.

**Figure 4:**
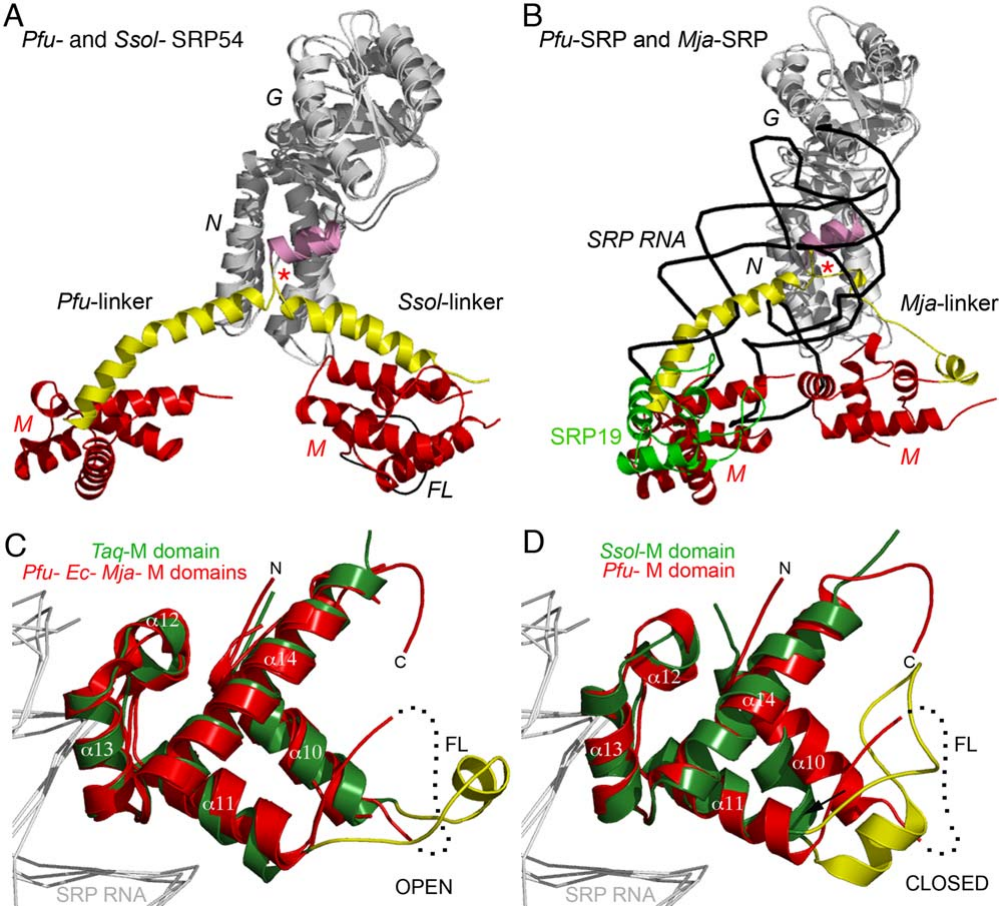
Conformational variability of the different Ffh/SRP54 proteins. (A) and (B) Conformational variability of the linker in the Ffh/SRP54 GTPases. (A) Superposition of the *Pfu*- and *Ssol*- free SRP54 structures. The NG domains (in grey) have been superposed to emphasize the different conformation adopted by the M domains (in red) and the G to M linkers (in yellow). The C terminal helices α7 of the G domains are highlighted (in pink). (B) Superposition of the *Pfu*-SRP54 and the *Mja*-SRP emphasizing the clash between the *Mja*-SRP RNA•SRP19 and the *Pfu*-M domain. In both figures *Pfu*-SRP54 is shown in the same orientation. The position of the glycine residues acting as “pivot points” is indicated with a red asterisk. (C) and (D) Conformational changes in the M domain. (C) The *Pfu*-M domain is shown superposed with the M domain as observed in the *Ec*, *Taq* and *Mja* structures. (D) The *Pfu*-M domain is superposed with the *Ssol*-M domain. As a reference the backbones of the SRP RNA from *Ec*, *Mja* and *Ssol* are shown in white. α helices are labeled according to the secondary structure assignment of *Pfu*-SRP54. The arrows emphasize the rearrangement and shift in position for the helix α10 when *Taq*, *Ssol* and *Pfu* structures are compared. In both figures the *Pfu*-M domain is shown in the same orientation.

*Pfu*-SRP54 crystallized in the tetragonal space group P4_2_2_1_2 with two monomers in the asymmetric unit resulting in a fairly high solvent content of 69%; the two monomers are related by a non-crystallographic two fold symmetry axis (**Figure 3A**). Phasing at 3.3 Å resolution was performed using single wavelength anomalous dispersion of selenium combined with molecular replacement using the archaeal NG domains structures of *Ssol* and *Mja* as models (see **Material and Methods**). The initial electron density maps using this MR-SAD combined approach unambiguously showed the NG to M domain linker (**Supplementary** **Figure S1**) allowing us to confidently trace the whole protein chain at this moderate resolution. The final 2.5 Å resolution structure of the free full-length SRP54 from *Pfu* was obtained in presence of GDP and is the highest resolution reported so far for a free full-length SRP54 with its linker fully resolved in the electron density map (**Figure 3B**).

In the two structures of *Pfu*- and *Ssol*- SRP54, the essential RNA-binding GTPase adopts an extended conformation. However superposition of the two structures using the NG domain as the reference body reveals that the relative orientation of the linker and the M domain are completely different and unrelated (**Figure 4A**). Within each *Pfu*-SRP54 monomer they are no interactions between the M domain and the NG catalytic core. This is in contrast to the structures reported for *Ssol*-SRP54 [19] and *Mja*-SRP [20], where there are interactions between residues from the tip of the N domain and residues located in the M domain and its finger loop. The present conformation of *Pfu*-SRP54 is incompatible with the assembly of the full SRP in presence of SRP19 as depicted in **Figure 4B** where superposition of the NG domains of *Pfu*-SRP54 and *Mja*-SRP54 structure shows that *Pfu*-M domain clashes with the SRP RNA and SRP19 protein in the fully assembled *Mja*-SRP [20]. The articulation point between the NG core and the M domain is located at residues Gly295 and Gly297 strictly conserved in all Ffh/SRP54 sequences (**Figure 2A**). These residues are part of a conserved motif R_292_XL**G**X**G**D_298_ (the GM-linker motif using *Pfu* sequence numbering) present in all SRP54 sequences and located between the helix α7 at the C-terminal part of the G domain and the linker. These conserved glycine residues act as pivot points allowing the M domain to sample diverse conformation while it scans for signal sequences as they emerge from the ribosomal exit tunnel. The affinity of SRP for presecretory proteins has been shown to be dependent on nascent chain length [21], this is probably in part linked to the flexibility and length of the linker that enables the SRP to adapt its conformation to different nascent chains.

The different SRP54 structures from *Thermus*, *Sulfolobus*, *Methanococcus* and now *Pyrococcus* represent a sampling of the conformations adopted by SRP-GTPase. Biochemical solution studies using fluorescence resonance energy transfer [22]–[24] or chemical foot-printing techniques [25] have shown that Ffh from *E.coli* undergoes major conformational rearrangements upon interaction with its SRP RNA and its cognate receptor. In the tetragonal crystals of *Pfu*-SRP54 containing two monomers for a total solvent content of 69%, we cannot rule out the possibility that the configuration of the M domain relatively to the NG core is at least partially affected by crystalline packing interactions. This observation can indeed be made for all other crystal structures of free SRP54 reported so far [18], [19]. The crystallization conditions for *Pfu* and *Ssol* SRP54s are quite similar (high concentration of lithium sulfate as precipitating agent in acetate buffer at an acidic pH but no detergent in our case). Although as we mentioned earlier and in contrast with the SRP54 structure from *Ssol* there are no contacts established between the NG and the M domains within a monomer of *Pfu*-SRP54, analysis of the crystallographic contacts shows interactions between the NG domain and the M domain of a crystallographically symmetry-related molecule (**Supplementary** **Figure S2A**). The 26 Å distance measured between the end of the α7 C-terminal helix of the NG domain (residue Leu296) and the N-terminus of the M domain (Gly326) from the closest symmetry-related molecule is much shorter than the 44 Å measured within the same protein chain. Thus this other conformation suggested by the analysis of crystal packing interactions is plausible assuming rearrangement of the linker. The GM-linker conformational variability previously observed in the *Taq*, *Mja* and *Ssol* structures and extended by the present structure would support this relative re-arrangement of the NG and M domains. This alternative conformation however, would not allow interaction with the SRP RNA since it partially occludes the SRP RNA binding interface of the M domain (**Supplementary** **Figure S2B**) and does not display the similar contacts observed between the N domain and the M domain in the *Ssol*-SRP54 and *Mja*-SRP structures (**Supplementary** **Figure S2C**).

### Architecture and conformation of the M domain

The M domain of *Pyrococcus furiosus* appears as a stable and well-defined structural module with the exception of the finger loop (residues L346 to D363) that was not observed in the electron density maps. The domain is organized around helices α10 through α14. A groove flanked by helices α10, α11 and α14 and the finger loop constitutes the putative signal sequence binding site [18], [26]. The average atomic displacement parameter for the M domains is B∼85 Å^2^, considerably higher than the 55 Å^2^ observed for the NG domains; this intrinsic flexibility has been proposed to be important for the ability to recognize diverse signal sequences at different states of exit from the ribosome.

Disorder in the finger loop was also observed in the *E.coli* [27] and in the *Mja* M domain structures bound to the core of the SRP RNA [9], [20], [28]. The Ffh/SRP54 structures from *Taq* [18] and *Ssol* [18], [19] however, each revealed differently structured finger loops. In *Ssol*, the finger loop appears to be defined and collapsed inside the hydrophobic groove, thus its M domain adopts a “closed” state where the finger loop folds back in the signal-binding grove shielding it from the solvent. In *Taq* the hydrophobic groove of the M domain is not empty but instead occupied by the finger loop from a neighboring molecule and might also contain some detergent from the crystallization solution. This is referred as an “open” state, primed for signal sequence binding. To date several structures of M domains have been reported but none has been solved in presence of a *bona-fide* signal sequence although a human SRP54 M domain structure [26] suggested a possible mode of binding the signal sequence in an α-helical configuration. Cryo-electron microscopy structures of SRP-ribosome complexes [29], [30] have revealed different modes for binding signal sequences in SRP bound to a ribosome-nascent chain complex.

Superposition of all M domain structures available in presence or absence of SRP RNA show that the free *Pfu*-SRP54 M domain conformation is similar to the “open” state (**Figures 4C and 4D**). Structure overlay also suggests that the RNA acts as a rigid backbone scaffold supporting the flexible M domain. A mutagenesis and biochemical study performed on *E.coli* Ffh revealed a subset of mutations that abrogates the effect of the SRP RNA and results in targeting defects *in vivo* [31]. These mutations map to the GM linker and the finger loop in the putative signal sequence binding groove of the M domain of Ffh, the two distinctive regions of Ffh/SRP54 that are found in many different configurations among known structures. In the GM linker, mutations L301P and L303D (corresponding to positions Q300 and L302 in *Pfu*) diminished the rate of stimulated GTPase activity of the SRP•FtsY complex. In the M domain finger loop region, mutations L350D and L354D (corresponding to the non-observed finger loop positions I349 and I353 in *Pfu*) impaired SRP RNA-catalyzed Ffh•FtsY complex formation without notably affecting RNA binding. This region is thus likely to sense the occupancy state of the binding groove and induce structural changes to the rest of the structure, probably mediated through the SRP RNA and the GM linker.

### The nucleotide-binding site

*Pfu*-SRP54 was co-crystallized in presence of GDP, and the nucleotide is clearly identified in the binding cleft located in the G domain (**Figures 2B** **and** **5**). Our structure of *Pfu*-SRP54 is the first full-length SRP54 structure bound to GDP. *Taq* is the only other SRP54 GTPase whose GDP-bound structure is known [32], [33] and this is for the NG domain alone not the full-length protein. All conserved motifs implicated in nucleotide binding are fairly well defined in the electron density. The protein interacts with the nucleotide through an intricate network of hydrogen bonds involving residues from the conserved motifs I, II, IV and V.

**Figure 5:**
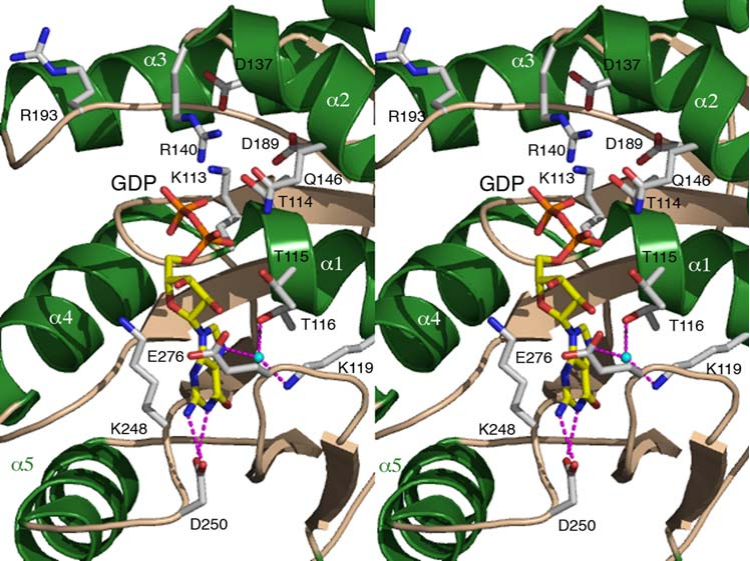
Stereo view showing the product GDP bound in the active site of *Pfu*-SRP54. All residues shown are strictly conserved in all SRP-GTPases. Helices have been labeled. Hydrogen bonds between the residue Asp250, the nucleotide specificity determinant, and the guanine ring are indicated. In contrast with one of the *Taq*-Ffh•GDP complex structures the sidechain of Arg193 is solvent exposed and does not establish a salt bridge with the Asp137 sidechain. This catalytic aspartate is located about 6.3 Å away from the GDP β-phosphate.

The paired hydrogen bonds between the carboxylate group of Asp250 in motif IV and the N1 and N2- nitrogens of the guanine ring are canonical nucleotide-specificity determinant seen in all SRP-GTPases [34], [35]. Interaction of this nucleotide specificity determinant also brings residues from the ‘closing loop’ (motif V) in close contact with the GDP. In particular, the sidechains from glutamate Glu276 and Lys248 in motif IV sandwich the guanine and ribose moieties of GDP. A water molecule mediates hydrogen bonding between the N7 nitrogen of the guanine ring and the sidechains of strictly conserved residues Thr116 and Lys119 in motif I. The α- and β- phosphates of GDP are held in place through hydrogen bond interactions with the insertion box domain (motif II) residues Arg140 and Gln146 and with residues Thr114 and Thr115 in motif I. These two residues also hold each other in place by hydrogen bonding. Lys113 sidechain from the P-loop sits between the carboxylate headgroups of aspartates Asp137 (motif II) and Asp189 (motif III) near the β-phosphate of the GDP but does not interact with the GDP. The catalytic Asp137 is located 6.6 Å away from the β-phosphate of the GDP. Despite the presence of magnesium in the crystallization liquour there is no clear sign of magnesium ion bound next to the GDP molecule. In the first *Taq*-FfhNG•Mg•GDP structure [32] this distance is 9.9 Å with Asp135 (Arg137 in *Pfu*) shifted away and establishing an ionic interaction with the strictly conserved Arg191 in motif II (Arg193 in *Pfu*). In *Pfu* however, residue Asp137 from motif II is brought closer (about 6.3 Å) to the nucleotide in a conformation and geometry similar to the one described in a more recent *Taq*-FfhNG•Mg•GDP structure [33]. Correspondingly to this shift in position the homologous ionic interaction is lost explaining why the arginine sidechain is solvent exposed.

### Structural coupling between the GTPase and the signal peptide-sensing domains

**Figure 6:**
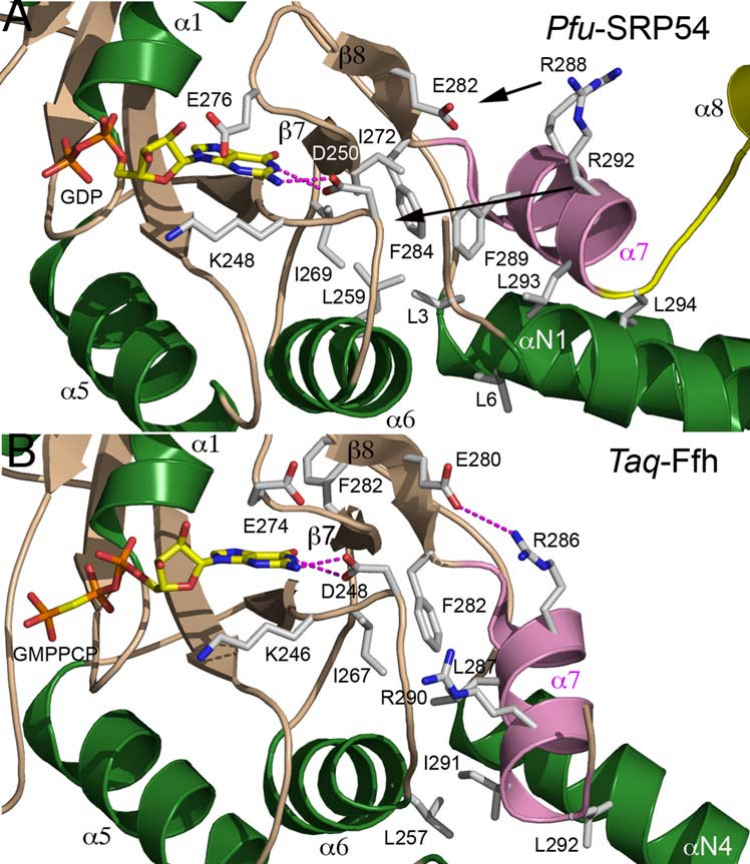
The GM linker couples the catalytic site of the GTPase and the signal peptide-binding domain. Based on the comparison between the *Pfu*-SRP54 GDP and *Taq*-FtsY•FfhNG•GMPPCP complexes, upon repositioning of helix α7, the basic residues Arg288 and Arg292 become buried at the NG interface: An ion pair forms between Glu282 and Arg288 and the sidechain of Arg292 interacts with and stabilizes the DARGG motif backbone. Rearrangement of a cluster of hydrophobic residues also propagates structural changes between the linker and the GTP binding site. The arrows emphasize the motions of all the conserved residues listed. (A) and (B) show the same area in the *Pfu*-SRP54•GDP complex and in the *Taq*-FtsY•FfhNG complex bound to GMPPCP.

In the *Pfu* structure, conserved residues Arg288 and Arg292 of the α7 anchoring-helix are well defined and solvent exposed (**Figures 3B** **and** **6A**) as in other structures of free full length Ffh (*Ssol* and *Taq*). In the *Mja*-SRP complex [20] the two equivalent side-chains (Lys288 and Arg292) are also externally oriented, pointing towards the SRP RNA backbone and may thus contribute to the docking of the NG domain against the RNA. By comparison, in the *Taq*-FtsY•FfhNG complex [36]–[38], [39], these two arginines are packed inside the protein core through a rearrangement of helix α7 (**Figure 6B**). In particular the positioning of the α7 helix is stabilized by two essential interactions: First, an interaction between the Arg290 sidechain (Arg292 in *Pfu*) and the DARGG motif links its position with the positioning of the conserved residue Asp248 (Asp250 in *Pfu*) that is directly involved in nucleotide recognition [40]; second an ion pair forms between Arg286 (Arg288 in *Pfu*) and Glu280 (Glu282 in *Pfu*). From analysis of the GDP-bound *Pfu*-SRP54 structure we can also hypothesize that rearrangement of a cluster of hydrophobic residues contributed by helices α6 (Leu259), α7 (Phe289 and Leu293) and the β-strands β7 (Ile272) and β8 (Phe284) will also affect the geometry within the catalytic site in response to structural changes occurring in the M domain and/or the SRP RNA. Repositioning of this basic ladder from the solvent exposed conformations, observed in the free SRP54 or entire SRP, to the buried conformation observed in the FtsY•FfhNG complex (**Figures 6A and 6B**) is likely to mediate communication between the signal peptide binding site in the M domain and the composite GTPase active site at the interface between the two G domains of Ffh/SRP54 and FtsY/SR.

The sequence motif RXLGXGD in the GM-linker motif appears to be a key structural element supporting the observed large domain rearrangements within the SRP54 and linking binding of external ligands (GTP and signal sequence) by SRP to the acquisition of the proper NG-M configurations required for the targeting process. It probably acts as a “sensor spring” and a lever able to transfer the conformational changes between the M and SRP RNA partners and the GTP catalytic center as they respectively interact with their cognate ligands (the signal sequence and the nucleotide).

### Free *Pfu*-SRP19 adopts a compact fold even in the absence of SRP RNA

**Figure 7:**
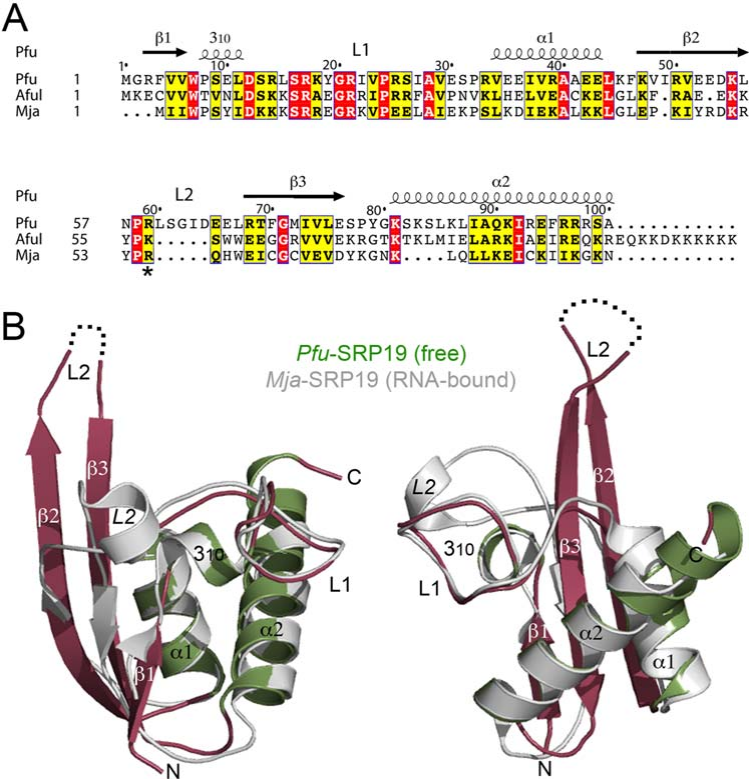
The SRP19 from *Pyrococcus furiosus*. (A) Sequence alignment of archeal SRP19 of known structures including *Pyrococcus furiosus*, *Archaeglobus fulgidus* and *Methanococcus jannaschii*. The secondary structure elements of *Pfu*-SRP19 are indicated. For the sake of clarity, the human SRP19 sequence [44] is not shown. (B) Two views of the monomer of free *Pfu*-SRP19 (in green and raspberry). The α helices, β strands and loops are labeled accordingly. The superposed structure of *Mja*-SRP19 as observed in the *Mja*-SRP complex is shown (in grey) to emphasize the overall rigidity of the protein backbones and the rearrangement of loop L2 upon binding to the SRP RNA. In free *Pfu*-SRP19 the L2 loop is disordered (dotted lines) whereas in *Mja*-SRP19 bound to SRP RNA it refolds and adopts an helical conformation.

We obtained two different crystals forms of free *Pfu*-SRP19 and determined their structures (**Table 1** **and** **Material and Methods**). Although an NMR structure of free SRP19 from *Archaeglobus fulgidus* [41] has already been reported, this is the first crystal structure reported for free SRP19. The protein adopts a βαββα fold (**Figures 7A and 7B**), similar to the K-homology (KH) domain and also resembles the RNP domain present in numerous and diverse RNA-binding proteins such as the single stranded RNA binding protein U1A [42] and the anticodon-binding domains of some aminoacyl-tRNA synthetases [43]. The structure is also characterized by two loops (L1 and L2). The loop L1, the primary RNA-binding surface is rigid and well defined in density whereas the loop L2 is disordered and absent in our final models. The disorder of loop L2 is also described in the NMR solution structure of free *Afu*-SRP19 [41]. *Pfu*-SRP19 is characterized by a more extended β-sheet due to longer β2 and β3 strands when compared with all other available SRP19 structures.

**Figure 8:**
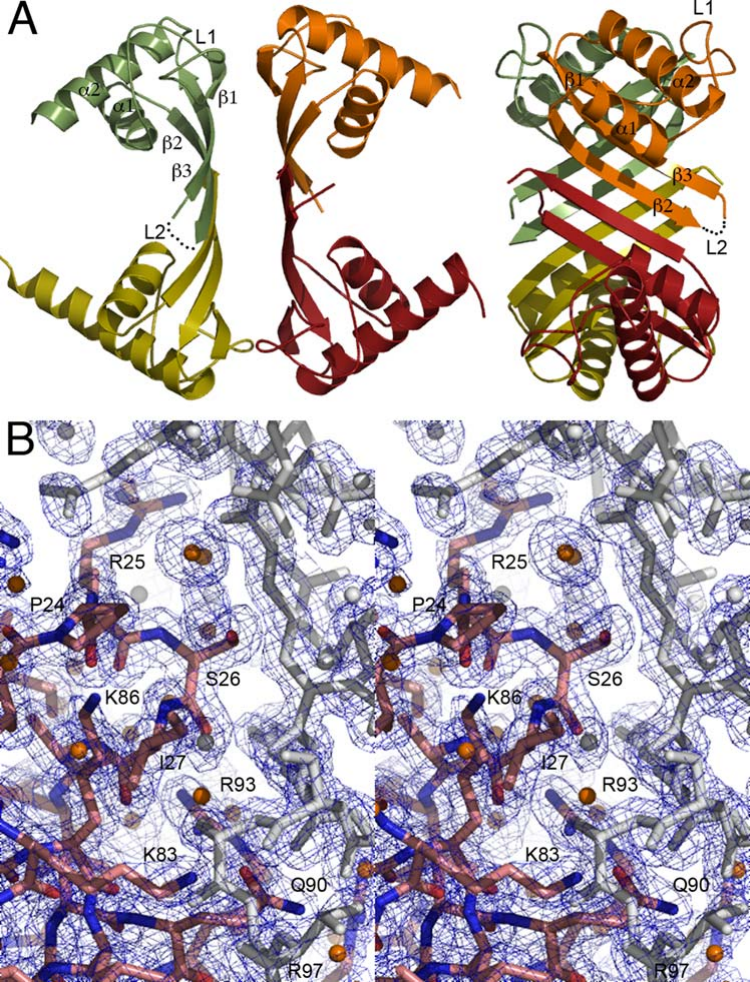
(A) Arrangement of the *Pfu*-SRP19 “tetramer” as observed in the asymmetric unit of the monoclinic crystal form. Two different orientations are shown. Each monomer is colored differently. The α helices, β strands and loops are labeled accordingly. (B) Stereo view of the 1.8 Å resolution *2mFo-DFc* Fourier difference likelihood-weighted electron density map contoured at 1.5σ in the loop L1 region of one monomer, water molecules are represented as spheres. For the sake of clarity symmetry related molecules are colored in grey.

**Figure 9:**
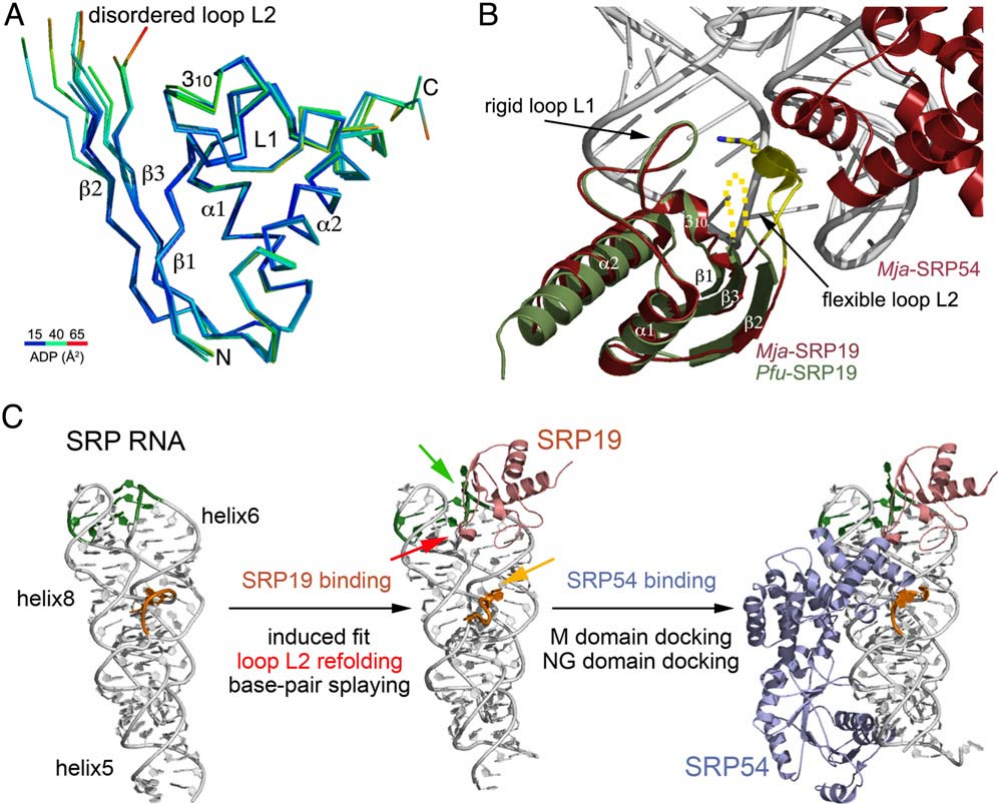
Closing of loop L2 upon SRP19 binding to the SRP RNA and its implications for the sequential assembly of the archeal SRP. (A) Superposition of the six crystallographically independent monomers of free *Pfu*-SRP19. The backbone trace is colored according to the atomic displacement factors. Dark blue corresponds to 15 Å^2^, green corresponds to 40 Å^2^ and red corresponds to 65 Å^2^. (B) Superposition of *Pfu*-SRP19 on the *Mja*-SRP19 as observed in the full *Mja*-SRP structure. *Pfu*-SRP19 and *Mja*-SRP19 are colored in green and red respectively. The *Mja*-SRP RNA is represented in white. The arrow emphasizes the rearrangement that the loop L2 is likely to undergo upon SRP19 binding to the RNA. (C) Model summarizing the role of SRP19 in the sequential assembly of the archeal SRP. The core of the archeal *Mja*-SRP RNA is shown, the nucleotides in the regions of the SRP RNA undergoing rearrangements during association are highlighted in green (primary SRP19 binding site) and orange (primary SRP54 binding site). As free SRP19 binds, through a reciprocal induced-fit mechanism, its disordered L2 loop folds (pink arrow). As was shown previously [9], the two SRP RNA regions where SRP19 (green arrow) and SRP54 (orange arrow) bind undergo concerted rearrangements, base pairs are splayed out and the RNA backbone is reconfigured resulting in a high affinity site for SRP54 M domain binding. Following docking of its M domain, the SRP54 NG domain may dock to the RNA backbone as observed in the *Mja*-SRP structure [20].

We solved two distinct crystal forms of *Pfu*-SRP19. In the monoclinic form the asymmetric unit contained a tetramer of SRP19 (**Figures 8A and 8B**) while the orthorhombic crystal form contained two molecules of SRP19. Two molecules of SRP19 associate in a dimer with an extended β-sheet surface built around the anti-parallel association of their respective β2 strands. The tetramer is organized around two dimers facing each other through their respective β-sheets at an angle of 45°. Observation of the crystal packing in the orthorhombic subunit reveals a tetrameric arrangement between symmetry-related molecules that is similar to the one observed in the monoclinic form. Superposition of all 6 independently refined monomers of *Pfu*-SRP19 show that the protein with the exception of the disordered loop L2 between the strands β2 and β3 is a rigid and compact structure (**Figure 9A**).

### SRP19 refolds upon binding to the SRP RNA: A reciprocally induced fit

The structures of SRP19 bound to SRP RNA are known in two organisms: The human SRP19 has been crystallized in presence of a short RNA fragment corresponding to the helix6 of the SRP RNA [44] and the SRP19 from *Mja* has been crystallized in presence of the full SRP RNA core composed of helices 6 and 8 and in presence or not of the M domain of SRP54 [28], [45], [46]. Superposition of our free SRP19 structure on the RNA-bound SRP19 structures reveals that the difference between free SRP19 and the RNA-bound forms lies exclusively in the conformation of the L2 loop region. The disordered L2 region of the “free” protein has a well-defined conformation in the RNA bound structures. In particular in the *Mja*-SRP structure, L2 rearrangement brings Arg55 (Lys59 in *Pfu*) in contact with the SRP RNA. As L2 folds, it not only establishes a conserved subset of interactions with the RNA but also some weak interactions with the M domain of SRP54/Ffh as observed in the more “complete” complex structures from *Mja* that also includes SRP54 [20] (**Figures 7B** **and** **9B**). In contrast the L1 loop remains structurally unchanged, this is observed even when all available structures of free and RNA-bound SRP19 from different species are compared. The L1 loop is the primary interaction surface of SRP19 on helix 6 of the SRP RNA. In the free *Mja*-SRP RNA [9], three unpaired bases directed toward the helical axis become inverted upon SRP19 binding and splay out in a conformation similar to the SRP54-bound form [20] (**Figure 9C**). Thus binding of SRP19 to the SRP RNA induces a structural change that facilitates the subsequent interaction of SRP54 and the proper assembly of the ribonucleic particle [28], [45], [46].

Time resolved foot-printing and fluorescence resonance energy transfer performed on the assembly of the human SRP revealed the existence of obligatory intermediates during the binding of SRP19 to SRP RNA [47], and showed that free human SRP19 is unstructured but forms a compact core upon binding to SRP RNA. Subsequent binding of SRP54 to the SRP19-RNA complex results in the assembly of an intimate tri-partite interface between SRP54, SRP19 and the RNA without significantly affecting the structure of SRP19 [48]. SRP19 is required in eukaryotes for the proper export of the SRP RNA from the nucleus to the cytoplasm [49] and for subsequent binding of SRP54 [50] and proper maturation and assembly of a functional SRP.

Our present work on the free *Pfu*-SRP19 supports the idea that this subunit acts as a molecular scaffold and a chaperone, assisting the SRP RNA in adopting the conformation required for its optimal interaction with the essential subunit SRP54 and thus ensuring the proper maturation and assembly of a functional SRP competent for protein-targeting through the interaction with its cognate receptor. Such mutual accommodation between protein and RNA-binding surfaces is a common theme in protein-nucleic acid interactions [51].

### Conclusions and Perspectives

We describe the X-ray structures of SRP54 and SRP19 the two protein constituting the proteinaceous core of the SRP from the hyperthermophilic archaeon *Pyrococcus furiosus*. Combined with our structure of *Pfu*-SR (*submitted*), this is to our knowledge the first case where all structures of the proteins constituting the SRP-dependent protein-targeting machinery from the same archeal organism are available. *Pfu*-SRP54 appears as a flexible molecule with a stable though dynamic M domain articulated on a flexible linker that connects it to the NG catalytic core. The linker region of SRP54 can adopt a variety of conformations that enable the signal peptide-binding domain to scan for diverse signal sequences as they emerge from the ribosomal protein synthesis exit tunnel and also regulates the activity of the GTPase core domain by coupling these two physically separated yet functionally interconnected domains. The structure of free *Pfu*-SRP19 reveals a compact entity and suggests that this subunit acts as a molecular scaffold and a chaperone involved in assembly of the functional ribonucleoprotein particle. Through an induced fit mechanism involving the rearrangement of its disordered L2 loop, SRP19 assists proper folding of the SRP RNA and therefore favors subsequent binding of SRP54.

SRP54 and SRP19 constitute the conserved core in all archaeal and eukaryotic SRPs. When compared with the somewhat simpler bacterial homologues, archaeal SRPs represent an increased level of complexity in terms of structural organization and mechanism of action. However, the intrinsic robustness and stability of their constituents in the case of thermophilic organisms may constitute an advantage. The archaeon *Pyrococcus furiosus* as a model system provides a platform suitable for further structural investigations of higher order complexes that can offer new insights into the mechanisms of the eukaryotic protein-targeting machinery.

## Materials and Methods

### Protein Expression and Purification

Genes encoding the full-length proteins *Pfu*-SRP54 (PF1731) and *Pfu*-SRP19 (PF1894) from *Pyrococcus furiosus* were amplified by PCR from total genomic DNA. The *Pfu*-SRP54 and *Pfu*-SRP19 genes were respectively cloned into the pET28b and pET29b vectors (Novagen) and the corresponding proteins expressed as fusions with either N- or C- terminal hexahistidine tags cleavable with thrombin. Native proteins were expressed in BL21(DE3)-*rosetta2 E.coli* cells grown in auto-induction media [52]. Seleno-methionine-containing proteins were expressed in B834(DE3)-rosetta2 *E.coli* cells grown in minimal media with glucose as carbon source and using the amino acid pathway starvation method [53], [54]. Purification of proteins was achieved in four steps combining heat selective precipitation, cobalt-chelating affinity chromatography, gel filtration and ion-exchange chromatography after removal of the purification tag. No detergent was used during purification or crystallization.

### Protein Crystallization

For crystallization, proteins were concentrated to 20 mg.ml^−1^ for *Pfu*-SRP54 and 15 mg.ml^−1^ for *Pfu*-SRP19. Crystals of *Pfu*-SRP54 and *Pfu*-SRP19 were obtained at room temperature from a variety of conditions in hanging drops by the vapor diffusion method using a Mosquito nanoliter-scale robotic workstation (TTP Labtech). Crystals of *Pfu*-SRP54 (tetragonal space group P4_2_2_1_2) grew in 1.0–1.3 M lithium sulfate and 100 mM sodium acetate pH = 5.0 with two monomers per asymmetric unit and a solvent content of 69%. Two crystal forms of *Pfu*-SRP19 were obtained in the same condition depending on whether the protein was native or seleno-labeled. Crystals of *Pfu*-SRP19 (monoclinic P2_1_ or orthorhombic C222_1_ space groups) grew in 1.2–1.3 M sodium malonate and 100 mM sodium acetate pH = 5.0. The monoclinic asymmetric unit contained four monomers for a solvent content of 42% whereas the orthorhombic asymmetric unit contained two monomers for a solvent content of 34%.

### X-ray Data Collection and Structure Refinement

X-ray diffraction data were collected at beamline 8.3.1 at the Advanced Light Source (Berkeley, California) on Quantum 210 or 315r CCD detectors. The structure of *Pfu*-SRP54 was solved using the anomalous dispersion signal of selenium at its excitation wavelength. The structure of the *Pfu*-SRP19 was solved using the anomalous dispersion of bromine at its excitation wavelength. The tetragonal crystals of *Pfu*-SRP54 cryo-protected in ethylene glycol diffracted to 2.5 Å. Both the monoclinic and orthorhombic crystal forms of *Pfu*-SRP19 were cryo-protected in ethylene glycol and diffracted to at least 1.9 Å resolution. Data were indexed, reduced and scaled with *HKL2000* [55] or *MOSFLM* [56] and *Scala* [57] using *Elves* [58]. SAD phasing, density modification and structure refinement were performed in *Phenix* [59]. Molecular replacement was done using *Phaser* [60]. Manual model building was done using *Coot* [61].

The *Pfu*-SRP54 structure was determined by MR/Se-SAD at 3.3 Å resolution and refined to 2.5 Å. The phasing process was improved through the combination of anomalous Patterson and Fourier differences, based on partial molecular replacement solutions obtained using various NG domain structures (from *Mja*, *Ssol* and *Taq*) as search models to locate seleniums and improve phases. These molecular replacement solutions were used to determine and verify the positions of the selenium sites in the NG domain based on sequence alignments. We did not use any of the available M domain structures. Initial phases were determined 3.3 Å after density modification, non-crystallographic symmetry averaging and solvent flattening in *Phenix*. Ten of the twelve expected selenium sites per monomer could be located unambiguously. Following initial location of the selenium scatterers, the figure of merit was 0.35 and was further improved to 0.67 after density modification. Inspection of the initial density map at 3.3 Å resolution allowed us to trace the linker between the NG and M domains without any ambiguity and revealed the presence of contaminating GDP bound to the protein at a low occupancy. The final 2.5 Å resolution native data set was collected on a crystal grown in presence of 10 mM GDP. Initial refinement cycles included non-crystallographic symmetry restraints that were removed at the final stage. The disordered finger-loop region encompassing residues Leu346 through Ile365 is missing in the final model.

The expression of seleno-substituted *Pfu*-SR19 proved inconsistent and attempts to grow crystals were particularly frustrating. Crystals of seleno-labeled protein were eventually grown in the same condition obtained for the native protein however they belong to the orthorhombic space group C222_1_ and despite the collection of a Se-SAD data set on a single crystal, we were unable to solve this structure using the observable anomalous signal. The *Pfu*-SRP19 structure (monoclinic form) was solved using a Br-SAD data set collected on a single native crystal briefly soaked in mother liquor supplemented with cryo-protectant and sodium bromide [62]. A total of 39 bromide ions were located in *Phenix* with refined occupancies ranging from 0.62 to 0.26. After initial location of the bromide scatterers, the figure of merit was 0.46 and was further improved to 0.67 after density modification. We eventually solved and refined the orthorhombic crystal form of *Pfu*-SRP19 by molecular replacement using the structure obtained with the monoclinic crystal form. Both structures were refined without non-crystallographic symmetry restraints. The disordered loop L2 region encompassing residues Asn57 through Glu66 is missing in the final models. Structure quality was assessed using *MolProbity* [63].

## Supporting Information

Figure S1 Experimental phasing of SRP54. Stereo view of the initial unbiased likelihood-weighted electron density map after MR-SAD phasing and density modification at 3.3 Å resolution contoured at 1.5σ showing the linker region between the NG and M domains. The backbone trace of the final model, refined against the 2.5 Å resolution native data set, is shown placed in density. The aN1 helix of the N domain, the α7 C-terminal helix of the G domain and the G to M linker region of helix α8 are labeled and colored in green, pink and yellow, respectively. The backbone positions of residues Arg288, Arg292, Gly295 and Gly297 are indicated. Arginines Arg288 and Arg292 constitute the so-called “basic ladder” and Glycines Gly295 and Gly297 are the pivot residues involved in the relative positioning of the M and NG domains. For the sake of clarity the trace of a symmetry-related molecule is not represented.
(2.49 MB TIF)

Figure S2 Analysis of crystallographic contacts between the NG and the M domains. (A) Overall arrangement showing the NG domain of one monomer (in salmon) and the GM-linker and M domain of its symmetry-related molecule (in blue). In this relative configuration, the distance between the end of the NG domain (Leu296 at the C-terminus of helix α7) and the N-terminus of the M domain (Gly326 at the C-terminus of helix α9) is 26 Å; the two boxed areas correspond to the only contact surfaces between the G and M domain (upper box) and the N and the G-M linker (lower box). (B) and (C) Close- up views of the two main contact areas. Residues involved in hydrogen bonding, van der Waals or ionic interactions are labeled. a-helices have been numbered.
(2.94 MB TIF)
