# Supplementary figures and images for: Structures of SRP54 and SRP19, the Two Proteins that Organize the Ribonucleic Core of the Signal Recognition Particle from Pyrococcus furiosus
Source: PLoS One. 2008 Oct 27;3(10):e3528. doi: 10.1371/journal.pone.0003528 (PMC2568955; doi:10.1371/journal.pone.0003528)

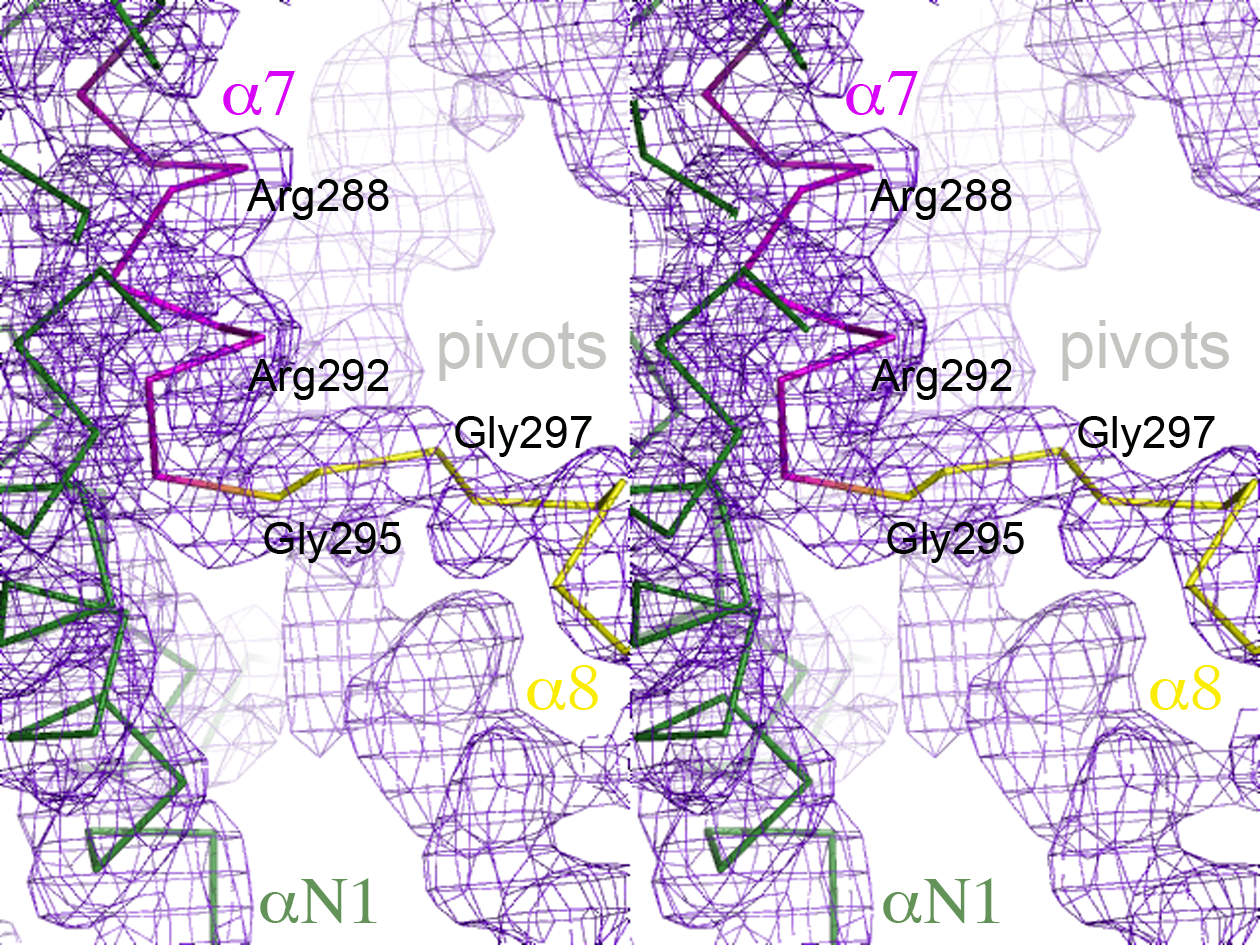

Supplement: Figure S1 — Experimental phasing of SRP54. Stereo view of the initial unbiased likelihood-weighted electron density map after MR-SAD phasing and density modification at 3.3 Å resolution contoured at 1.5σ showing the linker region between the NG and M domains. The backbone trace of the final model, refined against the 2.5 Å resolution native data set, is shown placed in density. The aN1 helix of the N domain, the α7 C-terminal helix of the G domain and the G to M linker region of helix α8 are labeled and colored in green, pink and yellow, respectively. The backbone positions of residues Arg288, Arg292, Gly295 and Gly297 are indicated. Arginines Arg288 and Arg292 constitute the so-called “basic ladder” and Glycines Gly295 and Gly297 are the pivot residues involved in the relative positioning of the M and NG domains. For the sake of clarity the trace of a symmetry-related molecule is not represented. (2.49 MB TIF) [file pone.0003528.s001.tif]

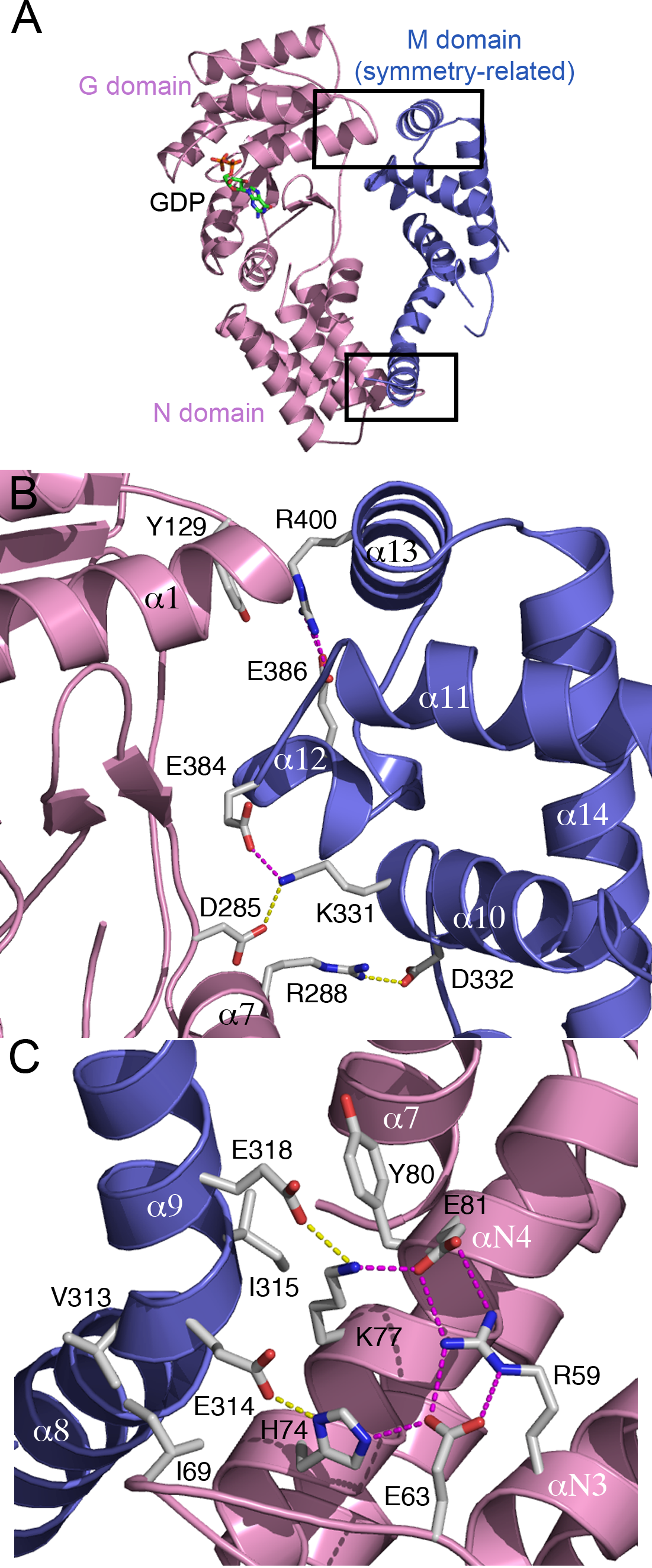

Supplement: Figure S2 — Analysis of crystallographic contacts between the NG and the M domains. (A) Overall arrangement showing the NG domain of one monomer (in salmon) and the GM-linker and M domain of its symmetry-related molecule (in blue). In this relative configuration, the distance between the end of the NG domain (Leu296 at the C-terminus of helix α7) and the N-terminus of the M domain (Gly326 at the C-terminus of helix α9) is 26 Å; the two boxed areas correspond to the only contact surfaces between the G and M domain (upper box) and the N and the G-M linker (lower box). (B) and (C) Close- up views of the two main contact areas. Residues involved in hydrogen bonding, van der Waals or ionic interactions are labeled. a-helices have been numbered. (2.94 MB TIF) [file pone.0003528.s002.tif]
